# Corrosion current density prediction in reinforced concrete by imperialist competitive algorithm

**DOI:** 10.1007/s00521-014-1645-6

**Published:** 2014-06-19

**Authors:** Lukasz Sadowski, Mehdi Nikoo

**Affiliations:** 1 Faculty of Civil Engineering, Wroclaw University of Technology, Wybrzeze Wyspianskiego 27, 50-370 Wrocław, Poland; 2 SAMA Technical and Vocational Training College, Islamic Azad University, Ahvaz Branch, Ahvaz, Iran

**Keywords:** Steel reinforced concrete, Resistivity, Polarization, Imperialist competitive algorithm, Genetic algorithm, Artificial neural networks

## Abstract

This study attempted to predict corrosion current density in concrete using artificial neural networks (ANN) combined with imperialist competitive algorithm (ICA) used to optimize weights of ANN. For that reason, temperature, AC resistivity over the steel bar, AC resistivity remote from the steel bar, and the DC resistivity over the steel bar are considered as input parameters and corrosion current density as output parameter. The ICA–ANN model has been compared with the genetic algorithm to evaluate its accuracy in three phases of training, testing, and prediction. The results showed that the ICA–ANN model enjoys more ability, flexibility, and accuracy.

## Introduction

Reinforced concrete (RC) is one of the most commonly used construction materials in civil engineering industry, and reinforcement corrosion is a major problem. For this reason, inspection techniques are used to evaluate steel corrosion in concrete in order to protect RC structures [1–9].

**Fig. 1:**
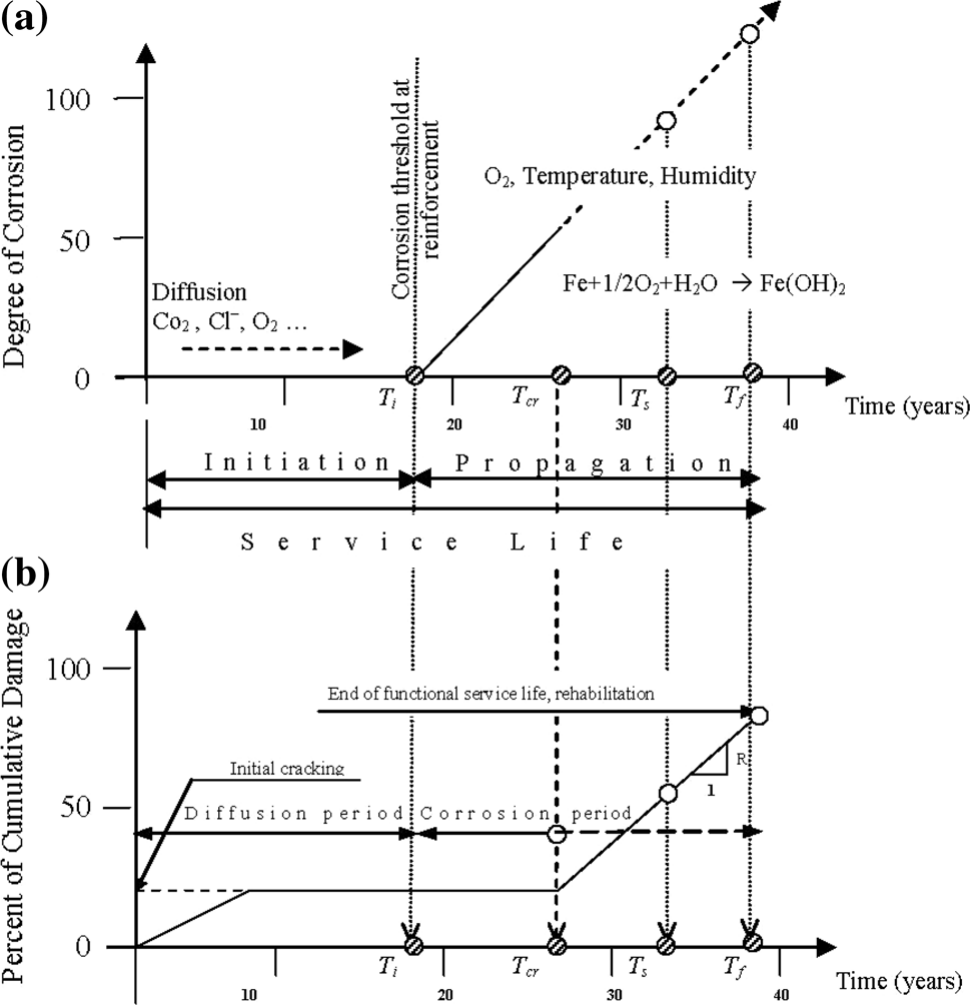
Models for corrosion of steel reinforcement in concrete: **a** model for predicting the service life of reinforcing steel [10], **b** performance-based service life model [11]

Tuutti developed the model for predicting the service life of reinforcing steel [10]. According to this model, the corrosion process has two distinct stages: corrosion initiation and corrosion propagation (Fig. 1a). Once this process has started, the time until damage occurs will mostly depend on relative humidity, the availability of oxygen, and temperature. When corrosion is initiated, active corrosion results in a volumetric expansion of the rust around the reinforcing bars against the surrounding concrete [10]. In the corrosion initiation stage, the required chemical reactions take place in concrete cover to initiate corrosion process. These chemical reactions may be carbonation or chloride ion attack.

Liu and Weyers [11] developed a performance-based service life model presented in Fig. 1b, which uses structural engineering performance criteria: serviceability and strength limit states. In service life model, the corrosion process represents three distinct phenomena: depassivation, propagation, and final state. Depassivation is the loss of oxide layer over the rebar due to the high alkalinity of concrete. Depassivation takes an initiation period *T*_i_ (time from completion of the new built structures to the time of corrosion initiation in the structure). The second life cycle is the propagation period *T*_s_ from the initiation of corrosion to corrosion-induced unserviceability of the structure. The third life cycle is the time period *T*_f_ from loss of serviceability to ultimate failure [12–15].

**Fig. 2:**
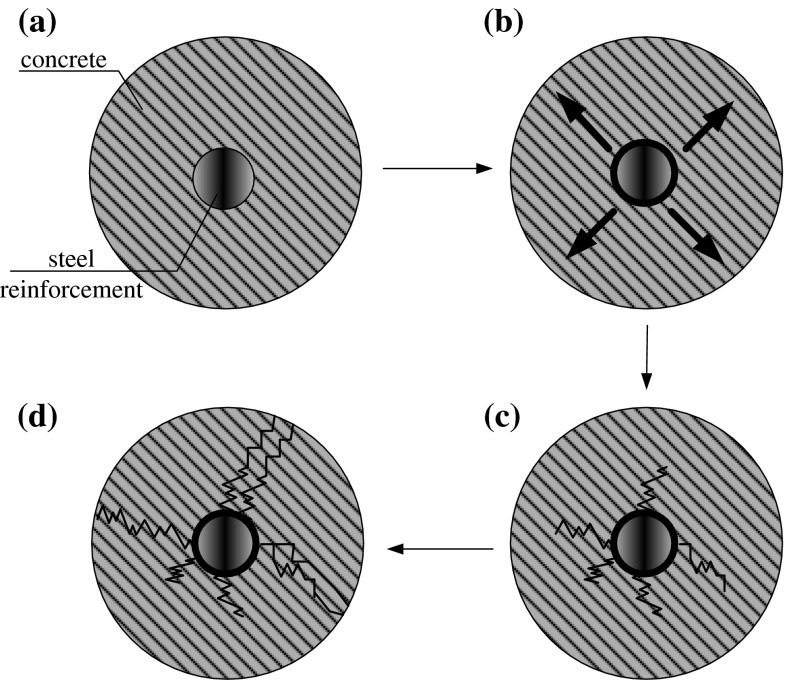
Deterioration mechanism of RC element as a result of corrosion process: **a** element prior to corrosion initiation, **b** expansive corrosion initiation, **c** crack propagation, **d** concrete cracking

It is proper to note that once the corrosion is initiated, concrete resistivity plays an important role in deciding reinforcement corrosion [16–18]. The concrete resistivity measurement provides additional information to assist in assessing corrosion process. As mentioned in [12], corrosion propagation is the phase in which the accelerated corrosion leads to rust staining, cracking of the concrete cover, and the deterioration mechanism in RC structures generally occurring in the form of longitudinal cracking, spalling, and delamination of concrete cover. Figure 2 shows the deterioration mechanism of RC element as a result of corrosion process.

One of the most useful methods of providing a direct evaluation of the corrosion rate by corrosion current density measurement *i*_corr_ is using the linear polarization resistance (LPR). In this method, a specific voltage shift (typically 10 mV) is applied to an electrode in solution. As mentioned in [16], the instantaneous corrosion current density *i*_corr_ is obtained by dividing a Stern–Geary [20] constant *B* by the polarization resistance *R*_*p*_ value:

1 $$i_{\text{corr}} = B/R_{\text{p}}$$

By measuring *i*_corr_, a corrosion rate can be derived. Typical corrosion rates from LPR measurements are presented in Table 1.

**Table 1:** Typical corrosion rates from LPR measurements [19]

Corrosion classification	Corrosion current density *i*_corr_ (μA/cm^2^)	Corrosion penetration rate^a^ (μm/year)
Passive/very low	Up to 0.2	Up to 2
Low/mod	0.2 to 0.5	2 to 6
Mod/high	0.5 to 1.0	6 to 12
Very high	>1.0	>12

^a^Loss of reinforcement section from Faraday’s Law, assuming Fe → Fe

LPR has its disadvantages because it can only be effectively performed in relatively clean aqueous electrolytic environments and in terms of destroying concrete lining to measure the amount of steel corrosion [21]. LPR will not work in gases or liquid emulsions where fouling of the electrodes will prevent measurements [22, 23]. It has been presented that corrosion current density is one of the most important input parameter in the corrosion-induced damage models [24]. Researchers developed corrosion current density prediction models based on the electrochemical principles of steel reinforcement corrosion [25], experimental testing [26], and statistical analysis [11].

It seems sensible to employ more parameters in one model to obtain a more accurate answer concerning the corrosion current density. Artificial neural networks (ANN) are massively parallel distributed processors that have a natural propensity for storing experiential knowledge and making it available for use [27, 28]. Nowadays, the ANN are well known and over the last few years have emerged as a powerful device that could be used in many engineering applications such for the prediction of corrosion of steel reinforcement in concrete [29–35]. ANN has been used, for example, as the prediction of chloride permeability of concretes with obtaining an empirical model having high capability of estimation of permeability for both their experimental data and the ones obtained from the studies of other researchers [36]. It has been also concluded that ANN model has a theoretical value in the prediction of the corrosion current rate of steel in concrete using corrosion current density without the need for a connection to the steel reinforcement [23].

## Research significance

**Fig. 3:**
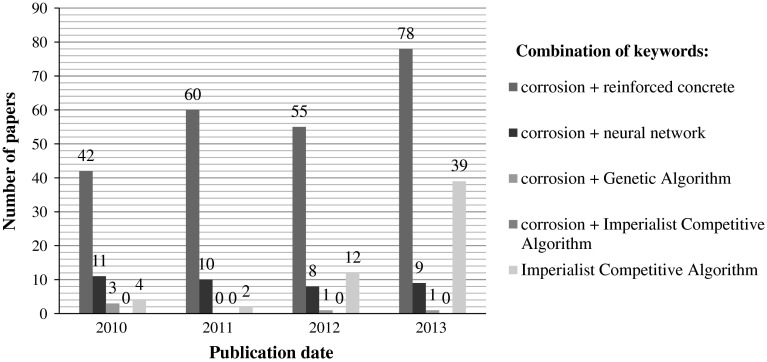
Number of papers related to corrosion of reinforced concrete published in Science Direct database since 2010

In previous research, backpropagation has been used for optimizing ANN. Although backpropagation has unquestionably been a major factor for the success of past ANN applications, it is plagued with inconsistent and unpredictable performances [37, 38]. Today, the new techniques exploiting ANN model, based on optimization algorithms, are being widely used in engineering fields [39]. The most popular seems to be imperialist competitive algorithms (ICA) and genetic algorithms (GA). The total number of papers related to corrosion of reinforced concrete published in Science Direct database increased from 42 in 2010 to 78 in 2013 (Fig. 3). The number of papers related to corrosion by using neural networks is on the same level since 2010. There were few attempts to use GA for the corrosion modeling. It is hard to find in the existed literature the application of ICA for corrosion current density prediction in steel reinforced concrete. It is proper to note that the applications of ICA for solving various engineering problems increased from 4 applications in 2010 into 39 in 2013.

ICA is a randomized population algorithm inspired from of the human political–social evolution [40–45]. A number of colonial countries along with their colonies try to find a general optimal point in solving optimization problem. Different methods have been introduced to solve optimization problems. Some find the cost function optimum point iteratively, based on the gradient. In spite of the high rate of these methods, there is still the problem of falling into the local optimum trap [46]. The main objective of this research is using ICA to optimize the weights of ANN as a new optimization algorithm in determining steel corrosion in concrete. The advantages of this method [46] have been listed below:

- The innovation of the ICA basic idea as the first optimization algorithm based on socio-political process;
- Ability to aligning and even higher optimization in comparing various optimization algorithms facing various optimization problems;
- Finding the optimal solution speed.

In the last few years, there were few attempts to apply ICA for the engineering problems modeling like for the prediction of soil compaction in soil bin facility [47], for the prediction oil flow rate of the reservoir [48], or for optimum cost design of cantilever retaining walls [49].

GA is inspired by nature. Nature evolution or Darwin’s theory is the basis of its formation in which the bests have the right to survival. In this method, chromosomes with high competence have a higher chance to repeat in the selected population in the replication process. This takes place by the selection process. Various methods have been proposed, and the wheel method is the most famous one. Also, the elitist selection is used to determine how many of the most graceful persons were transferred to the next generation, unchangeably [50, 51]. In the last few years, there were few successful attempts to apply GA for modeling the concrete structures such as finding optimum reliability-based inspection plans for the service life of the hypothetical bridge deck [52], finding optimal placements of control devices and sensors in seismically excited civil structures [53] or active control of high-rise buildings [54].

Considering the above in this paper, steel corrosion is determined and predicted in concrete using ICA–ANN model. Thus, the different aspects of the network will be checked with 2, 3, and 4 inputs; temperature, AC resistivity over the steel bar, AC resistivity remote from the steel bar, and the DC resistivity over the steel bar are as input parameters in the ANN, and corrosion current density is considered as an output parameter. The model ICA–ANN accuracy is evaluated compared with a GA, in three phases of training, testing, and prediction.

## Imperialist competitive algorithm (ICA)

**Fig. 4:**
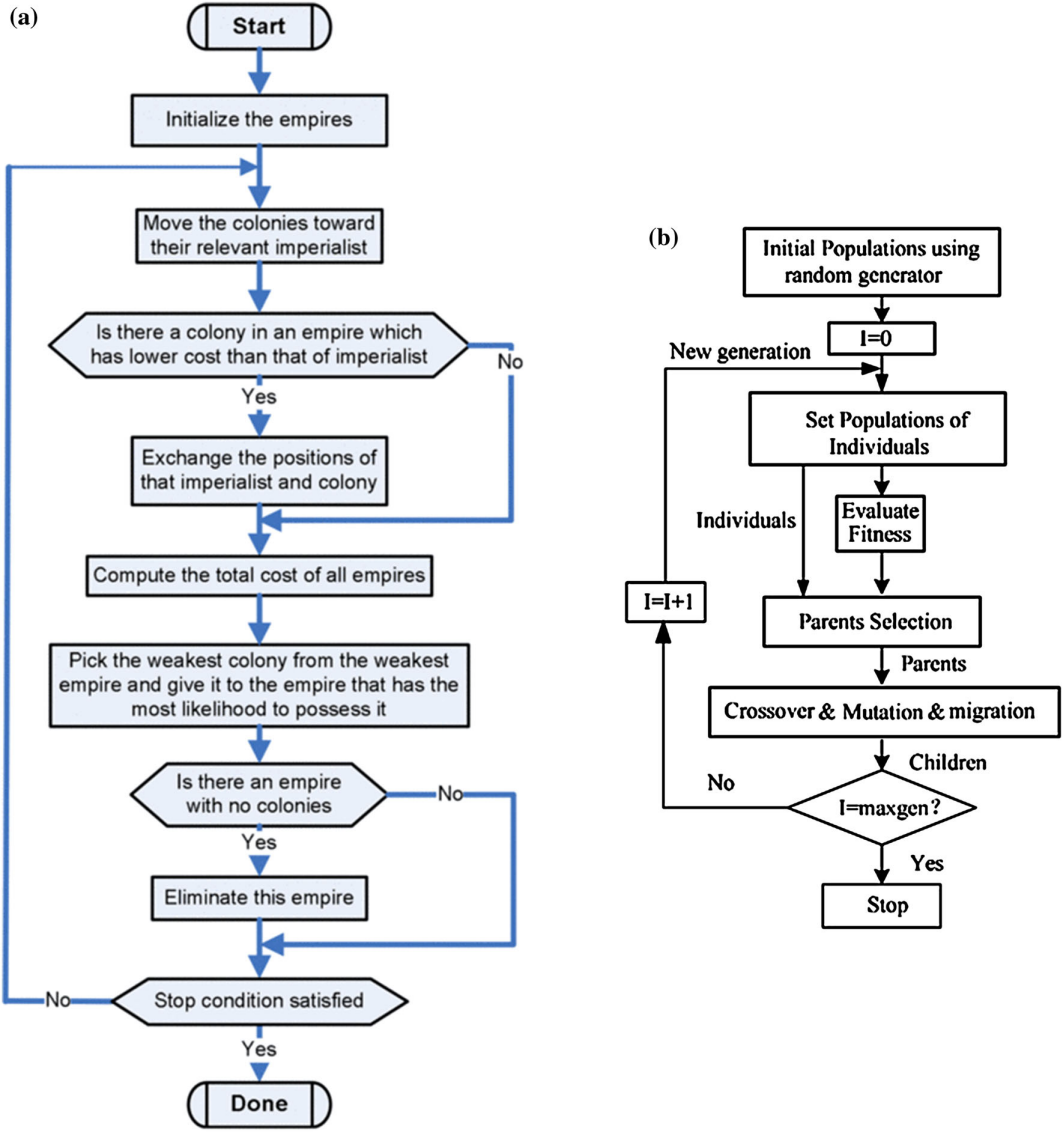
Flowcharts of: **a** ICA process [46], **b** GA process [60]

ICA is a method in the field of evolutionary computing that seeks to find the optimal solutions in various optimization issues, and it offers an algorithm for solving mathematical optimization problems. ICA forms an initial set of possible responses with a particular process improving the initial responses (countries) gradually and providing the appropriate response of optimization problem (the ideal country). The foundations of this algorithm are consisted of assimilation policies, imperialistic competition (IC), and revolution [46, 55]. ICA begins with random initial population, and some of the best elements of the population are selected as imperialists. The remaining population is considered as a colony. Depending on the colonial power, the colonies are absorbed by imperialists with a special process. The total power of the empire depends on both constituent parts, i.e., the imperialist country (as the central core) and its colonies [46]. This dependence has been modeled as the sum of the power of the imperialist country, plus a percentage of its average colonial power. With the formation of the early empires, IC between them will begin. Each empire unable to compete, succeed, and increase its power (or at least prevents reduction of its influence) will be removed in the IC. So the survival of an empire depends on its ability to absorb the colonial empires of the rivals and bringing them under control. As a result, during the IC, gradually, the power of larger empires will increase, and weaker empires will be removed. In Fig. 4a, the flowchart of ICA process has been presented [46].

Policy of assimilation was performed with the aim of analyzing the culture and social structure of colonies in culture of central government. Colonial countries, to increase their influence, began to build developmental infrastructures. In line with this policy, the colony will move as x units to the line connecting the colony to imperialist and will be drawn to a new position. X is a random number with uniform distribution. The distance between the imperialist and the colonized is shown by “*d,*” and the value of parameter *d* is shown by [46] in Eq. (2):

2 $$x\sim \, U\left( {0,\beta *d} \right)$$

where *β* is a number greater than one and close to two. A good choice can be *β* = 2. *β* ≥ 1 coefficient causes the colony country to near the imperialist country from different directions during moving to it. In addition to this move, a small angular deviation is added to the path with a uniform distribution [46]. Every empire unable to increase its strength and lose its competitiveness power will be removed in the IC. This process will be done gradually. This means that over time, weakened empires will lose their colonies and more powerful empire, will conquer them and increase their power—the empire being removed is the weakest one. Thus, when repeating the algorithm, one or more of the weakest colonies of the weakest empire will be taken, and to take possession of the considered colonies, a competition among all empires will be created. The colonies, not necessarily will be seized by the most powerful empire, but the stronger empires, are more likely to acquire them.

## Genetic algorithm (GA)

In this method, chromosomes with high competence have a higher chance to repeat in the selected population in the replication process. This takes place by the selection process [50]. After completion of the selection process, the operator is applied on the selected direction for reproduction. In the transplant process, with a constant transplant rate, a random number is generated for each chromosome. If the generated random number is less than the transplant rate, this chromosome is selected to intercourse the next chromosome with the above conditions. In this method, uniform transplantation has been used among different types of transplantation. Then, the mutation operator is applied [50]. The aim of this work is to create more dispersion in the range of design space. In the mutation process, a random number is generated, with a constant mutation rate, for all bits of the chromosomes. If the generated random number is smaller than the mutation rate, the value of that bit changes, i.e., the value of zero becomes one and vice versa. The basic operators of natural genetics are reproduction, crossover, and mutation. The basic operators of natural genetics are reproduction, crossover, and mutation [56, 57]. The GA can be expressed as in Fig. 4b. GA ends when certain criteria such as certain number of generation or the average standard deviation performance of individuals are satisfied [50, 58, 59].

## Experimental details and database

The data used for development of the models were obtained from the past experimental researchers [23, 61]. Exemplary steel corrosion data have been presented in Table 2. Of 68 data patterns, 80 % of samples (54 patterns) were used for training and 20 % of the selected samples (14 patterns) were used to test the network.

**Table 2:** Exemplary steel corrosion data

No.	*T* (°C)	*ρ*_AC,bar_ (kΩcm)	*ρ*_AC,coc_ (kΩcm)	*ρ*_DC_ (kΩcm)	*i*_corr_ (μA/cm^2^)
*Training*					
1	21.00	19.31	22.27	21.81	0.422
2	20.80	19.33	22.28	21.83	0.423
3	20.50	19.34	22.30	21.85	0.421
4	20.10	19.35	22.31	21.91	0.439
5	19.80	19.36	22.32	21.92	0.439
6	19.50	19.36	22.33	21.94	0.456
7	19.20	19.37	22.36	21.96	0.466
8	19.00	19.38	22.38	21.98	0.476
9	20.90	19.24	22.09	21.62	0.373
.	.	.	.	.	.
.	.	.	.	.	.
.	.	.	.	.	.
54	20.50	19.36	22.31	21.84	0.429
*Testing*					
1	20.10	19.34	22.33	21.91	0.434
.	.	.	.	.	.
.	.	.	.	.	.
.	.	.	.	.	.
14	19.10	19.30	22.22	21.77	0.421

**Fig. 5:**
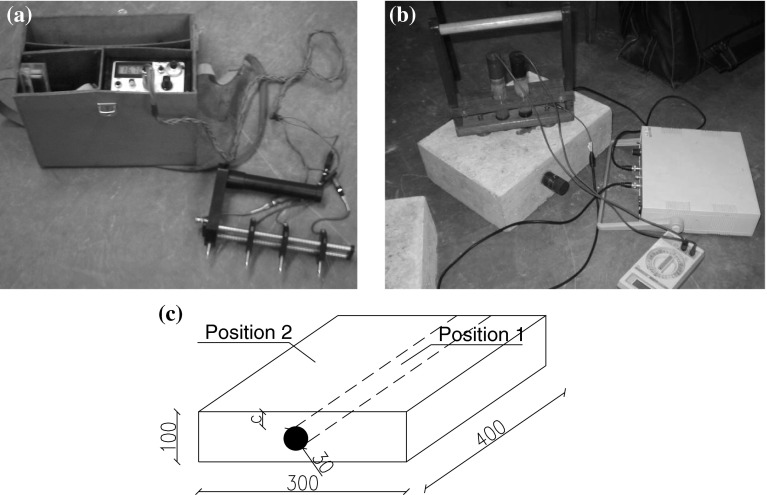
View of: **a** concrete resistivity measurement system, **b** laboratory stand, **c** resistivity measurement locations on concrete specimen [61]

Specimens sized 400 mm × 300 mm × 100 mm were available, with each specimen containing a single, short steel bar 30 mm in diameter, made from steel class A-III grade 34GS. The slabs were made from concrete class C 20/25 and from Portland cement CEM I 42.5R and aggregate of maximum size—5 mm. Since the relative humidity of concrete has a significant influence on the concrete resistivity, the specimens were stored in a laboratory under constant relative concrete humidity conditions of 65 ± 1 % up to the time of the tests. A view of the concrete resistivity measurement system, laboratory stand, and resistivity measurement locations on concrete specimen is presented in Fig. 5. The study was carried out based on a database of concrete slabs of two different conditions with high and moderate corrosion rates, respectively, with laboratory-induced corrosion as presented previously [61].

To include all the input parameters in a numerical range as the inputs of ANN to provide more accurate and suitable results, all data, according to formula (3), will be normalized:

3 $$xN = \left( {x - {\text{Min}}X} \right)/ \, \left( {{\text{Max}}X - {\text{Min}}X} \right) \times 2- 1$$

where *xN* are the normalized input data, xs are the input data, Min*X* is the minimum of all data, and Max*X* is the maximum of all the data.

Moreover, in the output parameter, the formula (4) will be used in normalization:

4 $$yN = \left( {y - {\text{Min}}Y} \right)/\left( {{\text{Max}}Y - {\text{Min}}Y} \right) \times 2- 1$$

where, *yN* are the normalized output data, *y*s are output data; Min*Y* are minimum of all data, Max*Y* are maximum of all data. Therefore, all normalized data should be located within numerical distance [−1, +1].

**Fig. 6:**
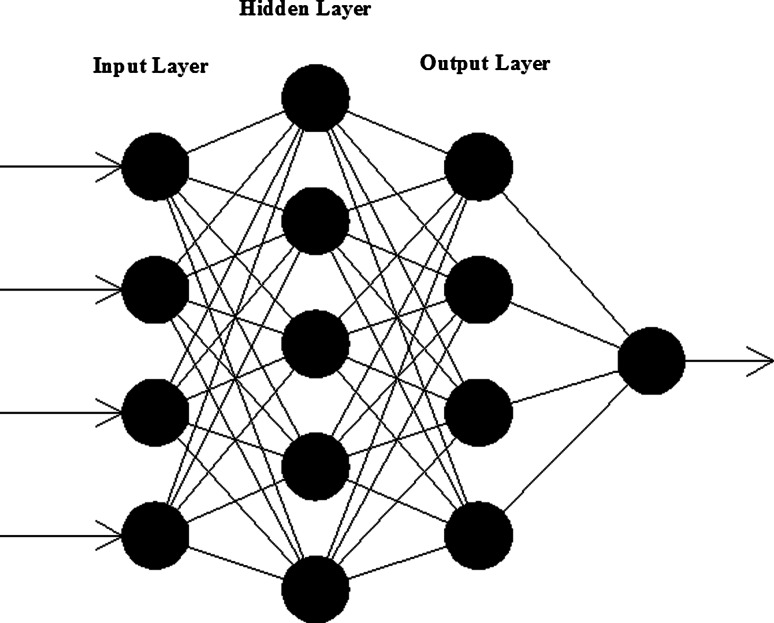
ANN with a hidden layer 4-5-4-1

ANN used in this research is called “Feed Forward” with the input parameters of temperature, AC resistivity over the steel bar, AC resistivity remote from the steel bar and the DC resistivity over the steel bar, and the output parameter corrosion current density; the network is shown in Fig. 6.

## Results

The hidden layer nodes numbers were determined through using [62] the empirical formula (5).

5 $$N_{H} \le 2N_{I} + 1$$

where *N*_*H*_ is the maximum number of nodes in the hidden layers, and *N*_*I*_ is the number of inputs. Considering that the number of effective inputs obtained is equal to 4, the maximum number of nodes in the hidden layer will be equal to 9. The ICA is used to determine the ANN model weights.

### Research conduction

Table 3 summarizes each pattern optima structure as well as the ICA features. Also, in Table 4, analytical results obtained from the training and testing patterns with optimized structure in Table 3 have been shown. In Table 5, three statistical parameters: mean absolute error (MAE), root mean squared deviations (RMSD), and root mean squared error (RMSE) have been applied.

**Table 3:** Optimized structure of ICA–ANN model

No.	Models’ name	ANN features					Utilized initialization parameters in ICA		
		Number of input	Number of output	Number of hidden layer	Number of nodes in hidden layer	Transfer function	Number of country	Number of imperialist	Number of decade
1	50GEN_2IN	2	1	2	3-2	tansig	300	20	50
2	50GEN_3IN	3	1	2	4-3	tribas	200	25	50
3	50GEN_4IN	4	1	1	9	satlins	400	40	50
4	100GEN_2IN	2	1	1	5	satlins	300	30	100
5	100GEN_3IN	3	1	1	7	tansig	400	40	100
6	100GEN_4IN	4	1	2	6-3	tansig	500	50	100
7	150GEN_2IN	2	1	1	5	poslin	400	20	150
8	150GEN_3IN	3	1	1	7	hardlims	250	25	150
9	150GEN_4IN	4	1	1	9	purelin	450	45	150
10	200GEN_2IN	2	1	1	4	tribas	600	60	200
11	200GEN_3IN	3	1	1	7	satlins	250	25	200
12	200GEN_4IN	4	1	3	3-3-3	poslin	500	50	200
13	250GEN_2IN	2	1	1	5	logsig	500	50	250
14	250GEN_3IN	3	1	2	4-3	radbas	250	25	250
15	250GEN_4IN	5	1	2	5-4	tansig	400	50	250

**Table 4:** Results from of testing and training of ICA–ANN model

No	Model	Best fitting line in testing phase		Best fitting line in training phase		Results		
		Equation	*R* ^2^	Equation	*R* ^2^	MSE test	MSE train	Best Cost
1	50GEN_2IN	*y* = 0.4714*x* − 0.1684	0.5487	*y* = 0.7753*x* − 0.0179	0.8043	0.1892	0.0482	0.0482
2	50GEN_3IN	*y* = 0.1945*x* + 0.1017	0.3751	*y* = 0.3443*x* + 0.174	0.5533	0.2709	0.2006	0.2006
3	50GEN_4IN	*y* = 0.5445*x* − 0.2061	0.4666	*y* = 0.9307*x* − 0.0289	0.7533	0.2332	0.0704	0.0704
4	100GEN_2IN	0.4734*x* − 0.1947	0.5391	*y* = 0.4734*x* − 0.1947	0.5391	0.2004	0.0452	0.0452
5	100GEN_3IN	*y* = 0.4282*x* − 0.1636	0.4646	*y* = 0.7518*x* − 0.0277	0.7866	0.2147	0.0523	0.0523
6	100GEN_4IN	*y* = 0.4578*x* − 0.1857	0.5922	*y* = 0.8676*x* − 0.0221	0.8752	0.1861	0.0303	0.0303
7	150GEN_2IN	*y* = 0.1746*x* + 0.0918	0.3745	*y* = 0.3095*x* + 0.163	0.5768	0.2760	0.2027	0.2027
8	150GEN_3IN	*y* = 0.7651*x* + 0.0221	0.21	*y* = 1.4463*x* + 0.2862	0.5108	0.8106	0.5836	0.5836
9	150GEN_4IN	*y* = 0.4931*x* − 0.1985	0.5176	*y* = 0.8187*x* − 0.0323	0.8231	0.2072	0.0430	0.0430
10	200GEN_2IN	*y* = 0.1761*x* + 0.0998	0.3862	*y* = 0.2953*x* + 0.1641	0.6067	0.2764	0.2059	0.2059
11	200GEN_3IN	*y* = 0.4827*x* − 0.1489	0.5297	*y* = 0.7894*x* − 0.0063	0.8273	0.1882	0.0430	0.0430
12	200GEN_4IN	*y* = 0.1397*x* + 0.0778	0.3724	*y* = 0.2625*x* + 0.1457	0.5857	0.2878	0.2084	0.2084
13	250GEN_2IN	*y* = 0.0752*x* + 0.091	0.4603	*y* = 0.1283*x* + 0.123	0.762	0.3230	0.2487	0.2487
14	250GEN_3IN	*y* = 0.2227*x* + 0.1323	0.3683	*y* = 0.3235*x* + 0.1799	0.5516	0.2711	0.2097	0.2097
15	250GEN_4IN	*y* = 0.7337*x* − 0.0618	0.8019	*y* = 0.8877*x* − 0.0042	0.9045	0.1572	0.0234	0.0234

**Table 5:** Statistical results of optimized ICA–ANN

No.	Model	MAE		RMSE		RMSD	
		Train	Test	Train	Test	Train	Test
1	50GEN_2IN	0.155	0.275	0.048	0.189	0.359	0.460
2	50GEN_3IN	0.309	0.394	0.201	0.271	0.475	0.559
3	50GEN_4IN	0.206	0.271	0.070	0.233	0.417	0.420
4	100GEN_2IN	0.410	0.490	0.264	0.360	0.586	0.635
5	100GEN_3IN	0.173	0.291	0.052	0.215	0.383	0.467
6	100GEN_4IN	0.410	0.490	0.264	0.360	0.586	0.635
7	150GEN_2IN	0.319	0.403	0.203	0.276	0.490	0.568
8	150GEN_3IN	0.660	0.726	0.584	0.811	0.765	0.772
9	150GEN_4IN	0.147	0.263	0.043	0.207	0.353	0.429
10	200GEN_2IN	0.331	0.410	0.206	0.276	0.513	0.584
11	200GEN_3IN	0.152	0.262	0.043	0.188	0.358	0.431
12	200GEN_4IN	0.335	0.421	0.208	0.288	0.512	0.588
13	250GEN_2IN	0.403	0.482	0.249	0.323	0.585	0.658
14	250GEN_3IN	0.320	0.388	0.210	0.271	0.486	0.558
15	250GEN_4IN	0.098	0.214	0.023	0.157	0.282	0.372

For models performance and determining the best model, the MSE train and MSE test criteria created by the models are compared with each other. According to the results shown, ANN model weights optimized by ICA with structure 4-5-4-1 and properties of 400 countries, 50 empires, and 250 iterations have been optimized, indicating the best results in the considered models.

As shown in Table 4 in the model No. 15, the coefficient *R* ^2^ for steel corrosion parameter in concrete in training and test phases is equal to 0.9045 and 0.8019, and also the slope of the straight line for this parameter is equal to 0.7337, 0.8877, which demonstrated the suitable accuracy of the model for modeling. Moreover, according to Table 5, MAE and RMSE and RMSD coefficients in both phases of training and testing of ANN with 4-5-4-1 structure and properties of 400 countries, 50 empires, and 250 iterations are lower than all models, which indicate less error of this network than other models. So ANN model under the title of 250GEN_4IN possesses higher accuracy than its peers.

**Fig. 7:**
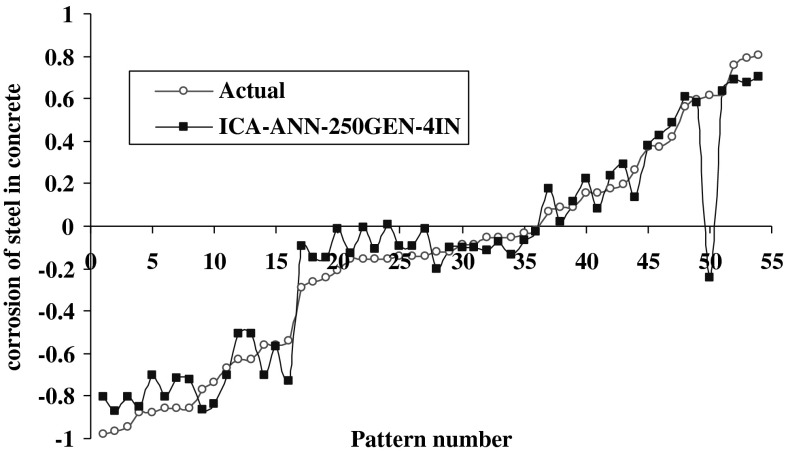
Determining corrosion current density in RC by ICA–ANN model in training phase

**Fig. 8:**
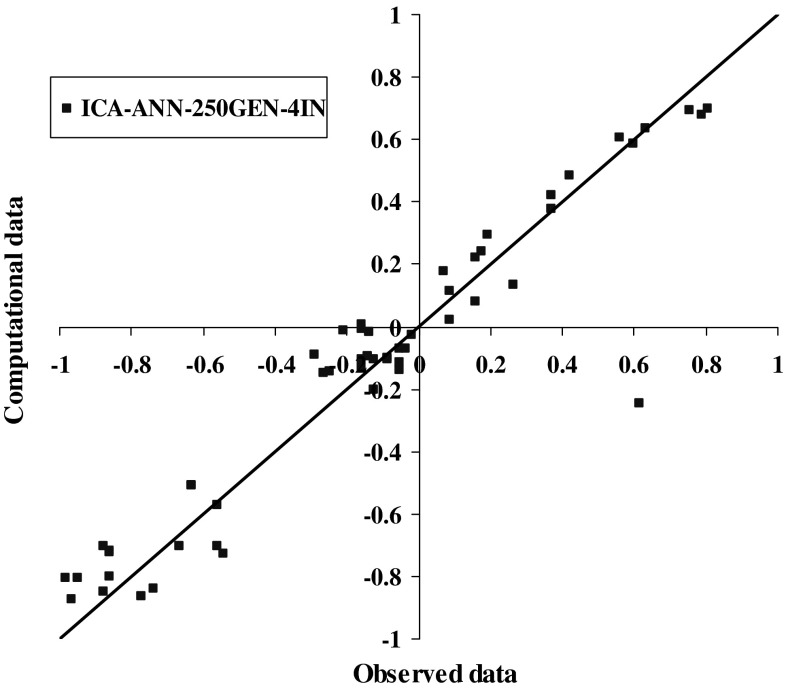
Comparing corrosion current density in RC by ICA–ANN model in training phase based on the observed data

**Fig. 9:**
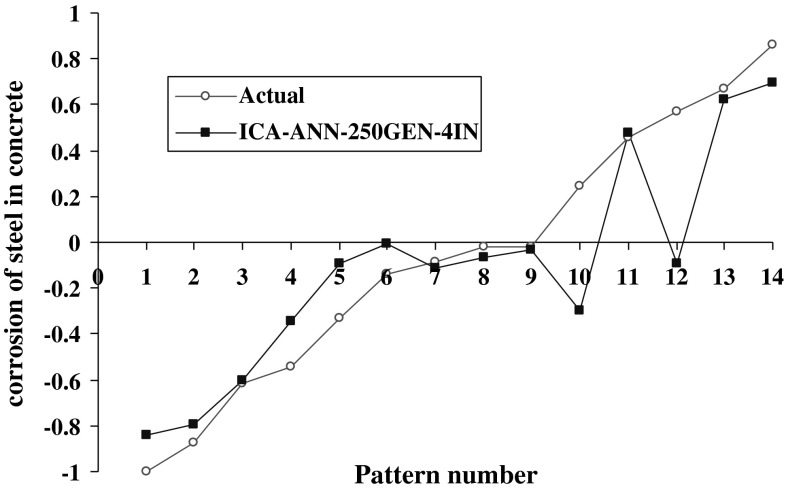
Determining corrosion current density in RC by ICA–ANN model in the test phase

**Fig. 10:**
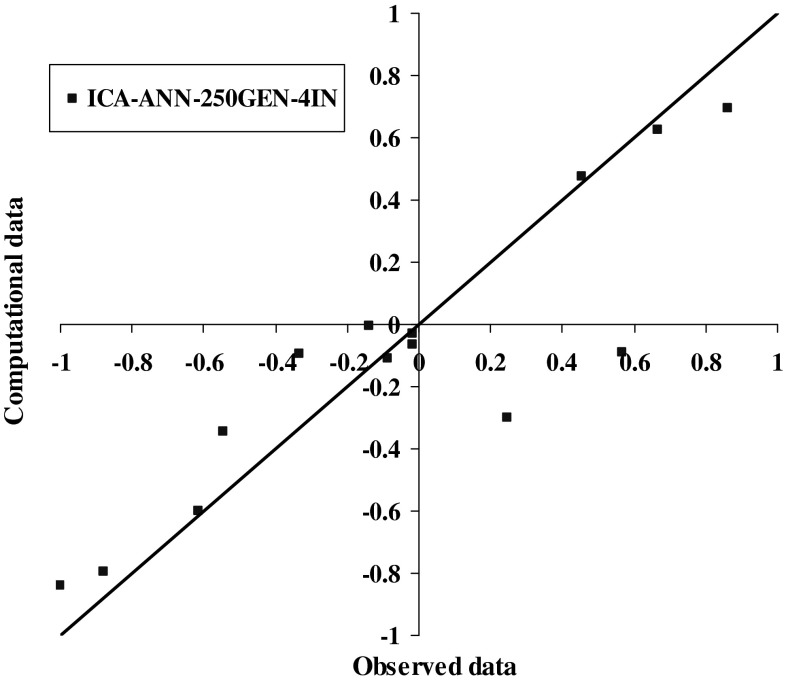
Comparing corrosion current density in RC by ICA–ANN model in the test phase based on observed data

**Fig. 11:**
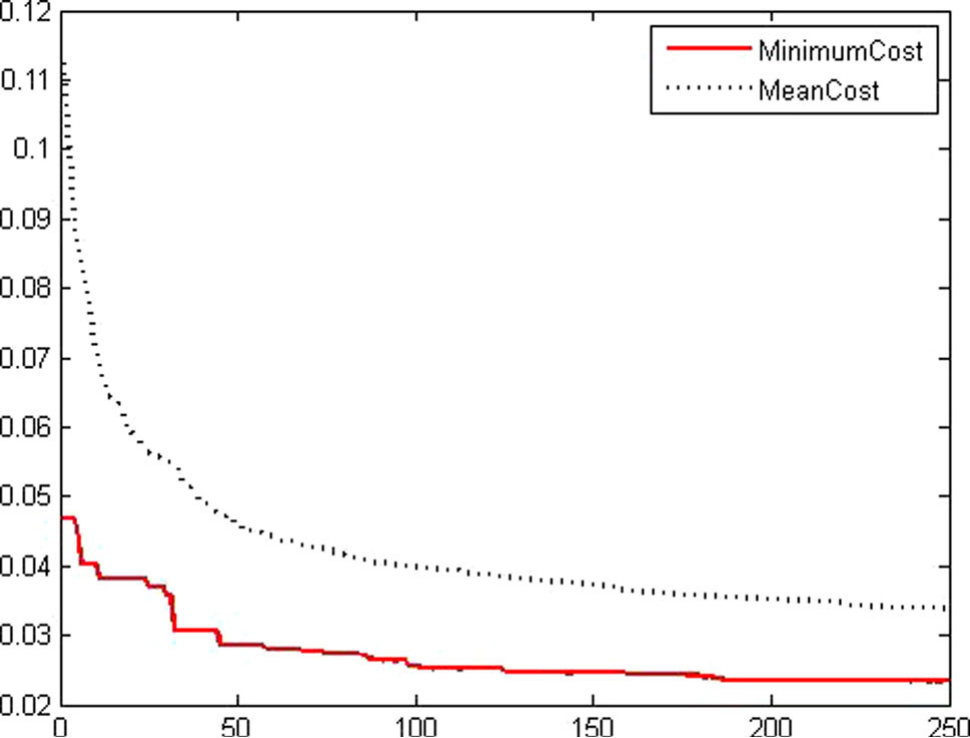
Graph cost for 250 iterations in the model FF-ICA-250GEN_4IN as the best model

The results of model 250GEN_4IN have been shown in Figs. 7, 8, 9, and 10 in comparison with observed data. The “mean cost” and the “minimum cost” curves are shown as the best models in Fig. 11. According to Fig. 10, “minimum cost” and “mean cost” coefficients are 0.0339 and 0.0234, respectively.

### Validating the model

To evaluate the accuracy of ICA–ANN model, it will be compared with GA. The properties of the models have been listed in Table 6. Besides, the ICA–ANN model with structure 4-5-4-1 and excitation function of Tansig have been exploited for two algorithms.

**Table 6:** Introducing ICA and GA parameters

GA		ICA	
Population	150	Countries	400
Mutation rate	15	Revolution rate	0.3
Crossover rate	50	Empires	50
		Uniting threshold	0.02
Generation	250	Generation	250

According to a survey conducted investigating training and testing data in the two models, *R* ^2^ coefficients and straight line slope have been shown in Table 6.

Table 7 shows the results of steel corrosion in concrete by the two. Table 7 discussed the results of the survey.

**Table 7:** Results of various algorithms in both training and test phases

No.	Model	Best fitting line in testing phase		Best fitting line in training phase	
		Equation	*R* ^2^	Equation	*R* ^2^
1	ICA–ANN	*y* = 0.7337*x* − 0.0618	0.8019	*y* = 0.8877*x* − 0.0042	0.9045
2	GA–ANN	*y* = 0.6239*x* − 0.1381	0.6618	*y* = 0.8098*x* − 0.0181	0.8505

**Fig. 12:**
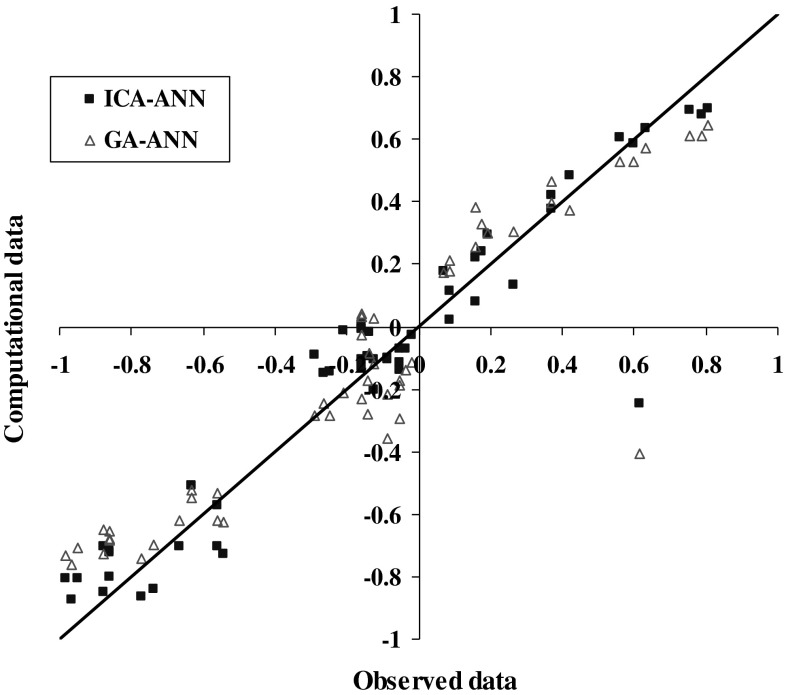
Comparing steel corrosion values in observational and computational concrete using algorithms ICA, GA during the training phase

**Fig. 13:**
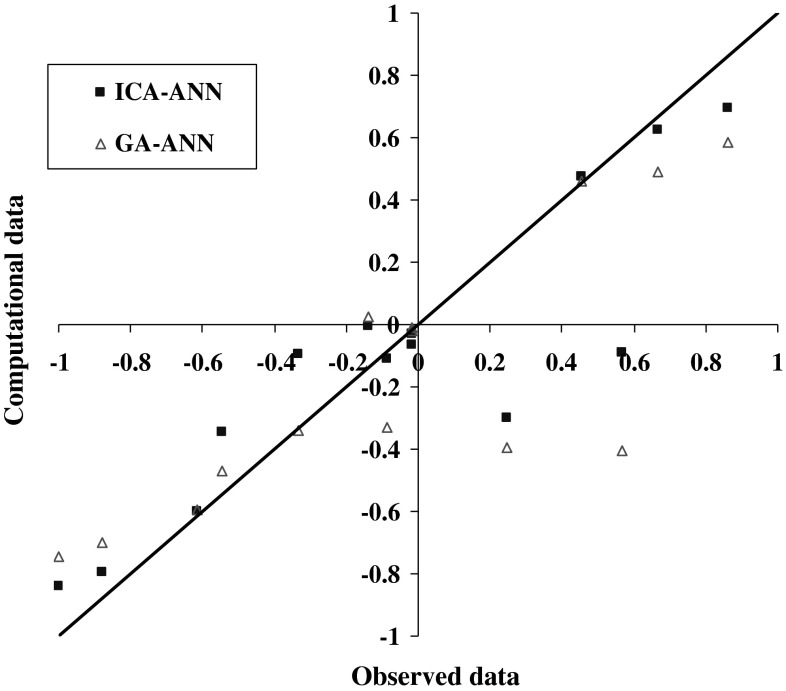
Comparing steel corrosion values in observational and computational concrete using algorithms ICA, GA in the test phase

Figures 12 and 13 show the comparison results of steel corrosion in computational and observational concrete by the two algorithms in the training phase. The cost graph is also shown by two algorithms in Fig. 13.

Due to the fitted lines equations presented on the computational and observational values in each model and determination coefficient related to each in Table 8, as well as the obtained results presented statistically in Table 7, it is clear that the ANN optimized by competitive ICA specifies steel corrosion much more carefully compared with GA. Given the determining of steel corrosion by two models in Figs. 11, 12 and 13, it is obvious that the ANN optimized by ICA is more accurate and flexible than the GA.

**Table 8:** Statistical results of ANN models optimized by ICA and GA in training and testing phases

No.	Model	MAE		RMSE		RMSD	
		Train	Test	Train	Test	Train	Test
1	ICA–ANN	0.098	0.214	0.023	0.157	0.282	0.372
2	GA–ANN	0.130	0.247	0.037	0.192	0.326	0.392

**Fig. 14:**
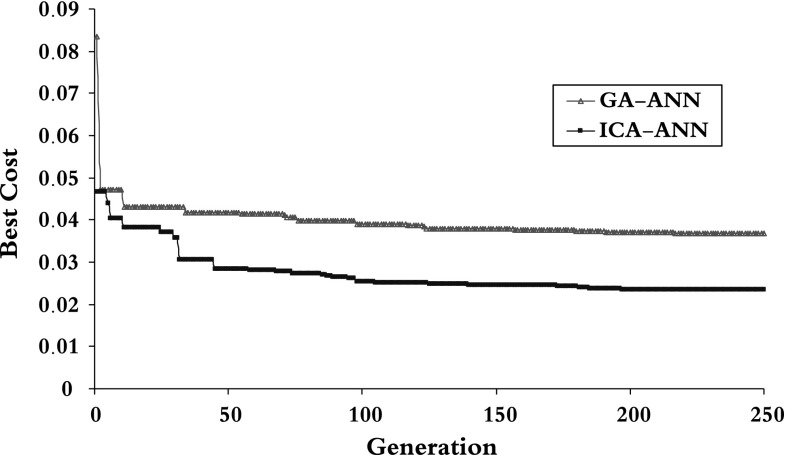
Cost graph of 250 iterations in three models ICA–ANN, GA–ANN

The cost graph has also been presented for the two models in Fig. 14.

To assess the optimum performance of ICA–ANN model in predicting the steel corrosion, its results are compared with GA–ANN results. Thus, 5 samples were used to predict, in two models. The main advantage of the models is that training and testing phases have not been used in any of them. Steel corrosion in concrete predicted by the two models has been listed in Table 9.

**Table 9:** Steel corrosion in concrete predicted by ICA–ANN and GA–ANN models

Row	Model	Results obtained by the graphs in training phase		Statistical parameters being evaluated		
		*R* ^2^	Equation	RMSD	RMSE	MAE
1	ICA–ANN	0.8087	*y* = 0.6836*x* − 0.0735	0.510	0.110	0.287
2	GA–ANN	0.7028	*y* = 0.5985*x* − 0.1396	0.517	0.174	0.328

**Fig. 15:**
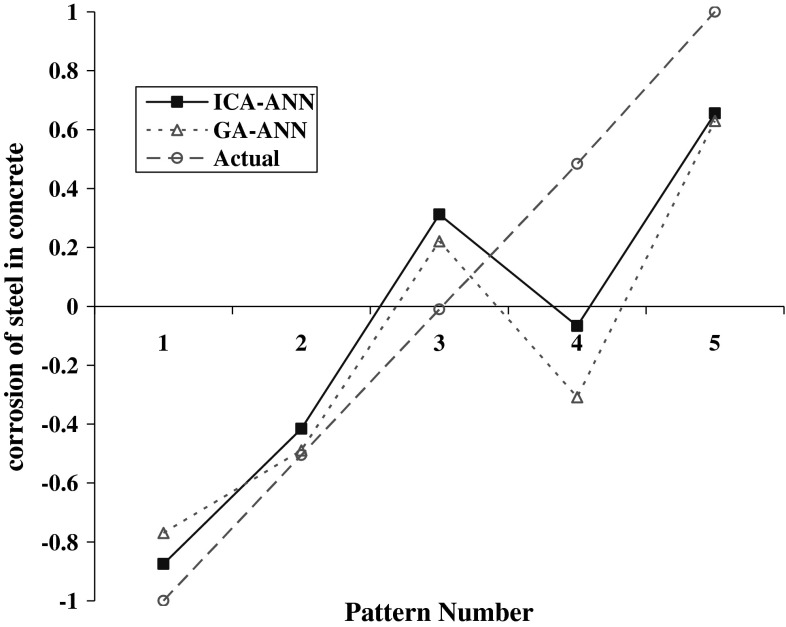
Predicting steel corrosion in concrete using various algorithms

**Fig. 16:**
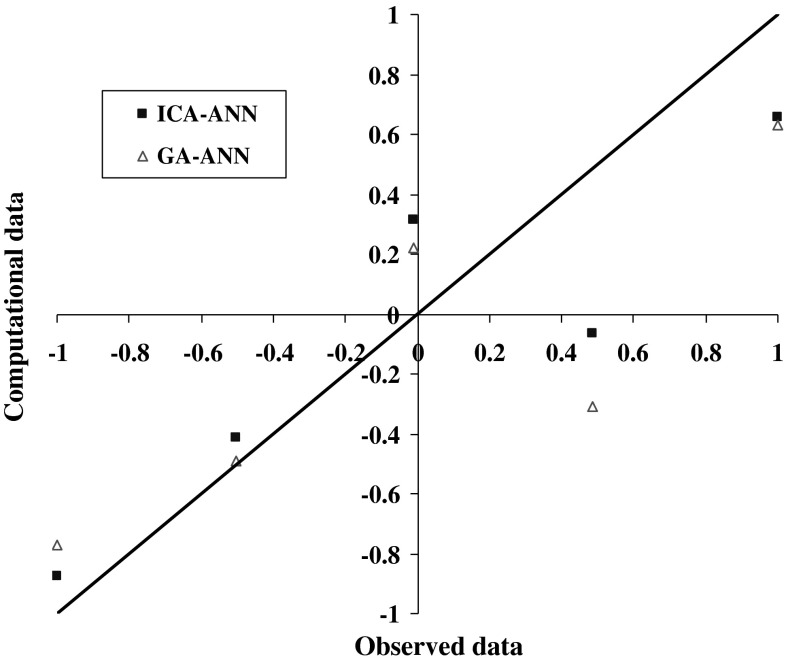
Comparing steel corrosion values in concrete for observational data and computational models in the prediction phase

Figures 15 and 16 provide comparing prediction of steel corrosion values in concrete for in terms of obtained results from calculations and actual data by the two models.

According to the presented fitted lines equations on steel corrosion rate in concrete in each model in Fig. 15 and the associated determination and statistical coefficients in Table 8, it can be concluded that the ICA–ANN evolutionary ANN model possesses more accuracy in prediction compared with GA–ANN model. Moreover, due to predicting steel corrosion in concrete by the two models in Fig. 14, we can conclude that ICA–ANN model is more accurate and flexible than GA–ANN model.

## Conclusions

A new proposition of corrosion current density prediction of reinforced concrete specimens subjected to corrosion test is presented in this numerical study. The proposed formulations are derived from the most popular algorithms used in ANN, namely ICA and GA. For this, available experimental data presented in the existing literature were gathered and used for prediction. Based on the analysis of the results, the following conclusions can be drawn:

- The experimental results of the proposed method on the ANN architecture showed that the ICA is quite successful in finding the functions’ optimum point. The practical problems solved with this algorithm also demonstrated that the proposed optimization strategy can be successfully applied along with other techniques such as GA and particles swarm to help solving practical problems. Comparing the results obtained by the proposed algorithm with the optimization conventional methods indicated the relative superiority of this algorithm.
- The ability to optimize the ICA can be used as a powerful tool to optimize ANN weights.
- Comparing the results of training and testing ANN different models optimized using ICA, indicated that the ANN with 4-5-4-1 structure, the transfer function of Tansigmoid and the associated properties to Colonial Competitive Algorithm with 400 countries and 50 primary emperors and 250 iterations, is able to accurately predict corrosion current density in RC.
- In the best ANN optimized by ICA in predicting steel corrosion rate in concrete in the training and test phase, the coefficient is 0.8019 and 0.9045, respectively, and also the line slope of this parameter is equal to 0.7337 and 0.8877, indicating the higher accuracy of model to the peers, also in this model, the statistical factor values of MAE, RMSE and RMSD are less than all models, which presented lower error in this model.
- Results showed that the ANN optimized with ICA possesses higher accuracy and flexibility in predicting steel corrosion in concrete than GA.

## Recommendations

The purpose of this paper is to predict corrosion current density in concrete using ANN combined with ICA used to optimize weights of ANN. The ICA–ANN model has a theoretical value and potential practical significance in the prediction of the corrosion current rate of steel in concrete using corrosion current density without the need for a connection to the steel reinforcement, and it may help the engineers in practice to make a comparison for the corrosion performance of steel in concrete in RC elements. However, to increase the generalization capability, the database should be further enhanced by increasing the number of training samples, a wider range of concrete humidity, corrosion current density, or the concrete’s resistivity.
